# HTLV-1 and HTLV-2: highly similar viruses with distinct oncogenic properties

**DOI:** 10.3389/fmicb.2014.00398

**Published:** 2014-07-29

**Authors:** Vincenzo Ciminale, Francesca Rende, Umberto Bertazzoni, Maria G. Romanelli

**Affiliations:** ^1^Department of Surgery, Oncology and Gastroenterology, University of PaduaPadua, Italy; ^2^Department of Life and Reproduction Sciences, University of VeronaVerona, Italy

**Keywords:** HTLV, Tax, Rex, HBZ, APH2, clonality

## Abstract

HTLV-1 and HTLV-2 share broad similarities in their overall genetic organization and expression pattern, but they differ substantially in their pathogenic properties. This review outlines distinctive features of HTLV-1 and HTLV-2 that might provide clues to explain their distinct clinical outcomes. Differences in the kinetics of viral mRNA expression, functional properties of the regulatory and accessory proteins, and interactions with cellular factors and signal transduction pathways are discussed.

## INTRODUCTION

Four types of HTLV have been described so far, the most prevalent types being HTLV-1 and HTLV-2 (Gessain et al., 2013).

HTLV-1 and HTLV-2 show considerable homology in terms of genome structure, replication pattern, and properties of the structural, regulatory, and accessory proteins. Their transmission follows the same route, by transfer of infected lymphocytes by perinatal transmission and breastfeeding and through blood transfusion, sexual contact, and use of intravenous drugs (Pique and Jones, 2012). Both viruses utilize the GLUT-1 and NRP1 cellular receptors for their entry, although HTLV-1, but not HTLV-2, is dependent on heparan sulfate proteoglycans (Jones et al., 2006). Cell-to-cell transmission is essential for virus replication and occurs through the formation of a virological synapse (Nejmeddine et al., 2005).

Despite these important analogies, HTLV-1 and HTLV-2 are strikingly different in terms of clinical impact, as only HTLV-1 is conclusively associated with neoplasia, namely adult T-cell leukemia/lymphoma (ATLL), which develops in up to 5% of infected individuals. A similar percentage of HTLV-1-infected persons develop a neurological disease termed HTLV-associated myeolopathy/tropical spastic paraparesis (HAM/TSP; Gessain and Mahieux, 2012). In contrast, HTLV-2, though persistently associated with elevated lymphocyte and platelet counts (Bartman et al., 2008) and with an increase in overall cancer mortality (Biswas et al., 2010), does not cause hematologic disorders and is only sporadically associated with myelopathy (Araujo and Hall, 2004).

The two viruses also differ in their geographical distribution. HTLV-1 is endemic in Japan, sub-Saharan Africa, South America, and the Caribbean (Gessain and Cassar, 2012), whereas HTLV-2 is prevalent among the indigenous populations in Africa and the Indian-American tribes in Central and South America as well as among drug users in Europe and North America (Zella et al., 1990; Roucoux and Murphy, 2004). Although their receptor usage allows HTLV-1 and HTLV-2 to be quite promiscuous for different cell types *in vitro*, they exhibit distinct cellular tropisms *in vivo*: HTLV-1 is mainly found in CD4+ T lymphocytes, whereas CD8+ T cells are the preferred target for HTLV-2 (Ijichi et al., 1992).

The following sections highlight differences in the biology of HTLV-1 and HTLV-2 that might provide clues to their distinct clinical outcomes. Emphasis is placed on the comparison of the regulatory proteins, kinetics of mRNA expression, clonal distribution patterns, and interaction with cellular factors and signal transduction pathways.

## THE COMPARATIVE ANATOMY OF THE HTLV-1 AND HTLV-2 GENOMES

In their pioneering studies, Seiki et al. (1983) noted the presence of a region in the HTLV-1 genome between the env open reading frame (ORF) and the 3′ LTR that was not present in the previously described oncogenic retroviruses. This region, termed the “X region,” is also present in HTLV-2 and codes for the regulatory and accessory proteins in partially overlapping ORFs named x-I through x-V. Expression of the complex arrangement of ORFs in such a compact genome is accomplished by a combination of ribosomal frameshifting, alternative splicing, and polycistronic translation (Ciminale et al., 1992, 1995; Koralnik et al., 1992) as well as production of negative-strand transcripts that code for the HBZ (HTLV-1) and APH-2 (HTLV-2) proteins.

Like other retroviruses, the HTLVs produce an unspliced mRNA which codes for the Gag-Pol-Pro precursor protein and also serves as the viral genome and a singly spliced mRNA that codes for the Env surface glycoproteins. Peculiar to the HTLVs is the expression of the Tax and Rex regulatory proteins that play critical roles in driving expression from the 5′ LTR promoter (Tax) and in enhancing the expression of partially spliced plus-strand mRNAs (Rex). The Tax and Rex ORFs (x-IV and x-III, respectively) are expressed from the same doubly spliced mRNA. In HTLV-1, all the other X region proteins are coded by individual singly or doubly spliced transcripts, while some HTLV-2 transcripts express more than one ORF.

Recent studies of the temporal sequence of HTLV-1 gene expression in peripheral blood mononuclear cells (PBMCs) isolated from infected patients (Corradin et al., 2011; Rende et al., 2011) revealed a “two-phase” kinetics with tax/rex mRNA expression preceding that of other viral transcripts. A similar analysis of HTLV-2 mRNA expression indicated a comparable pattern, although the relative abundance of some transcripts showed some intriguing differences among the two viruses (Bender et al., 2012; Cavallari et al., 2013).

The major differences between HTLV-1 and HTLV-2 gene products are summarized in **Table 1** and commented in the following sections.

**Table 1:** Structural and functional differences between HTLV-1 and HTLV-2 gene products.

Structural and functional properties	Viral proteins		Reference
	HTLV-1	HTLV-2	
	**Tax-1**	**Tax-2**	
LZR (leucine zipper domain)	+	-	Higuchi and Fujii (2009)
p100 binding	+	-	Shoji et al. (2009)
PDZ domain	+	-	Suzuki et al. (1999)
CD4 + immortalization	+	++	Imai et al. (2013)

	** p21Rex**	**tRex**	
Inihibition of full-length Rex	-	+	Ciminale et al. (1997) Bai et al. (2012)

	**p30Tof**	**p28**	
Interaction with the co-activator CBP/p300	+	-	Zhang et al. (2001)

	** p13**	**-**	
Increases mitochondrial K+ permeability and ROS production; inhibits Tax function in the nucleus	+	-	D’Agostino et al. (2005) Silic-Benussi et al. (2009) Andresen et al. (2011) Silic-Benussi et al. (2010b)

	**p12**	** p10**	
Binding MHC-I heavy chains	+	+	Johnson et al. (2000)
Binding to the IL-2R, calreticulin and calnexin and NFAT activation	+	-	Mulloy et al. (1996) Ding et al. (2001) Ding et al. (2002) Albrecht et al. (2002)

	**p8**	**-**	
Increases T-cell contacts and intercellular conduits	+	-	Van Prooyen et al. (2010b)

	-	**p11**	Ciminale et al. (1995)

	**Antisense viral proteins**	
	**HBZ**	**APH-2**	

Inhibition of Tax-mediated transcription	+++	+	Halin et al. (2009)
p300/CBP interaction	+	-	Clerc et al. (2008)
Transcriptional activity of c-Jun family	-	+	Marban et al. (2012)
Induction of cell proliferation	+	-	Douceron et al. (2012)
Binding to Tax	-	+	Hivin et al. (2005)
bZIP domain	+	-	Marban et al. (2012)

## Tax PROTEINS AND THEIR INTERACTIONS WITH CELLULAR PATHWAYS

The principal role of Tax during viral replication is to activate transcription of the LTR promoter through a process that involves the recruitment of CREB/ATF complexes on binding sites in the U3 region. Tax-1 and Tax-2 share this mechanism and are able to cross-activate each others’ LTRs (Semmes et al., 1996).

Experiments performed in the early 1990s established a key role for Tax as the main viral oncoprotein necessary for the initial steps of T-cell transformation by HTLV-1 (Tanaka et al., 1990). Transgenic mouse models have consistently demonstrated that Tax expression causes tumors, including lymphoma and leukemia (Ruddle et al., 1993; Hasegawa et al., 2006). Recent comparative studies on the transforming activities of HTLV-1 Tax (Tax-1) and HTLV-2 Tax (Tax-2) demonstrated that, in the absence of other viral proteins, both Tax-1 and Tax-2 immortalize human primary CD4+ T cells, but only Tax-2 is able to immortalize CD8+ T cells (Imai et al., 2013). Furthermore, Tax-2 immortalizes human primary CD4+ T cells more efficiently than Tax-1 (Imai et al., 2013). Although additional viral products have been demonstrated to be relevant for immortalization, the interactions between Tax and host proteins are still considered to be essential for the transformation process. Several studies of the oncogenic properties of Tax-1 highlighted its effects on DNA repair, cell cycle progression, cell death, and p53 function (reviewed by Cheng et al., 2012).

In addition to CREB/ATF proteins, Tax-1 interacts with many other host factors that influence cell proliferation and transformation, including components of the PI3K, AKT, MAPK, TGFβ, SRF, and NF-κB pathways (Romanelli et al., 2013). Tax-2 has been analyzed mainly for its effects on the NF-κB pathway, and was shown to activate the canonical pathway, but, unlike Tax-1, does not activate the noncanonical pathway (Shoji et al., 2009), suggesting that activation of this latter pathway might be a key element in HTLV-1-driven transformation *in vivo*. Although overall highly homologous, Tax-1 and Tax-2 present some distinct structural features. Tax-1 contains a PDZ binding motif (PBM) and leucine zipper domains that are absent in Tax-2. The C-terminal PBM domain is relevant for interaction of Tax-1 with host factors that regulate cell cycle progression and proliferation, such as the human homologue of the *Drosophila melanogaster* disk large tumor suppressor protein (hDLG; Suzuki et al., 1999). The leucine zipper regions present in the central region of Tax-1 are responsible for activation of the non-canonical NF-κB pathway (Higuchi and Fujii, 2009). The nuclear localization and nuclear export signals of Tax-1 and Tax-2 present significant differences which could explain the predominant nuclear localization of Tax-1 compared to Tax-2 (Turci et al., 2006, 2009; Avesani et al., 2010).

An additional difference between Tax-1 and Tax-2 that may play a role in NF-κB activation is the absence of interactions between Tax-2 and TNF receptor-associated factor TRAF6, an E3 ubiquitin ligase that is involved in ubiquitination of effectors of the NF-κB pathway (Journo et al., 2013). Both Tax-1 and Tax-2 are ubiquitinated and SUMOylated, but they differ in the pattern of modification (Zane and Jeang, 2012). The role of ubiquitination, sumoylation, and acetylation of Tax in NF-κB activation and cellular transformation is still an open field of research (Turci et al., 2012; Xiao, 2012; Journo et al., 2013; Lodewick et al., 2013).

Activation of NF-κB by Tax is also connected to the deregulation of autophagy, an additional pathway that is altered in oncogenic signaling. Both Tax-1 and Tax-2 induce autophagosome accumulation, but their interactions with the components of the process differ. In fact, Tax-1 (but not Tax-2) directs the IKK complex to lipid rafts associated with autophagic molecules such as Beclin 1 and Bif-1 (Huang et al., 2009; Ren et al., 2013).

## POST-TRANSCRIPTIONAL REGULATION BY Rex

HTLV-1 Rex (Rex-1) and HTLV-2 Rex (Rex-2) share 60% homology at the amino acid level. Rex-1 and Rex-2 are phosphorylated proteins that actively shuttle between the nucleus and the cytoplasm (Palmeri and Malim, 1996; Narayan et al., 2003) and accumulate in the nucleus and nucleoli (Nosaka et al., 1989; Ciminale et al., 1995, 1997; Nosaka et al., 1995; Narayan et al., 2003), a property that is intimately linked to their ability to enhance the nuclear export of incompletely spliced viral mRNAs.

Rex-1 and Rex-2 share a similar domain structure which includes: (i) a nuclear localization signal (NLS; Siomi et al., 1988; Nosaka et al., 1989), which mediates binding to the RXRE (Grassmann et al., 1991) located at the 3′ end of all HTLV-1 transcripts and at the 5′ end of the unspliced HTLV-2 mRNA (Ohta et al., 1988; Black et al., 1991); (ii) multimerization domains; and (iii) a leucine-rich sequence located near the middle of the protein (Rex-1 aa 79–99; Rex-2 aa 81–94), which functions as an activation domain (AD; Weichselbraun et al., 1992) and contains the nuclear export signal (NES; Kim et al., 1996; Palmeri and Malim, 1996) that interacts with CRM1/exportin, which mediates the nuclear export of the Rex-viral mRNA complex (Bogerd et al., 1995; Kim et al., 1996; Palmeri and Malim, 1996). In addition a C-terminal domain unique to Rex-2 is a target for serine phosphorylation and may also contribute to efficient nucleo-cytoplasmic shuttling (Narayan et al., 2003). Although Rex is not required for cellular immortalization *in vitro*, it is necessary for infectivity and viral persistence *in vivo* (Ye et al., 2003), as it is required for the expression of the virion-associated structural proteins.

The dependence of HTLV mRNAs on Rex function is controlled by positively and negatively acting RNA sequences present on the primary transcript. Two major types of such RNA *cis*-acting elements have been described: (i) the Rex responsive element (RXRE) which, besides binding Rex, acts as an inhibitory sequence in the absence of Rex, and (ii) *cis*-acting repressive sequences (CRS) that determine poor stability and/or inefficient nucleo-cytoplasmic export. HTLV-1 contains a CRS which maps at the 5′ end of the unspliced mRNA but is spliced out of the other transcripts (Seiki et al., 1990; King et al., 1998). An additional CRS overlaps the RXRE and acts synergistically with the 5′-CRS. In contrast to the 5′-CRS, this 3′-CRS/RXRE is present at the 3′ end of all viral transcripts. Both the 5′- and 3′-CRSs were shown to act mainly as nuclear retention sequences. The 5′-CRS does not bind Rex-1 (Ballaun et al., 1991; Bogerd et al., 1991; Unge et al., 1991), and its inhibitory function is likely to be mediated by other viral and/or cellular RNA-binding proteins. Other *cis*-acting inhibitory elements (CIEs) were mapped within the gag-pol and env regions of HTLV-1 (Saiga et al., 1997). The inhibitory effect of these regions is counteracted by binding of Rex-1 to the RXRE, although it is not clear whether they function mainly at the level of RNA stability or nucleo-cytoplasmic export. A 5′-CRS acting as a nuclear retention sequence was also mapped in the R-U5 region of HTLV-2 (Black et al., 1991).

## THE ACCESSORY PROTEINS

The “accessory” proteins were labeled as such because their ablation does not have apparent consequences on viral replication *in vitro*. However, studies performed in animal models indicate that some of these proteins are essential for efficient infectivity *in vivo*. Among the accessory proteins, p30Tof/p28, p12/p10, and p21Rex/tRex (in HTLV-1 and HTLV-2, respectively) are considered to be homologous based on their structure and functional properties, while p13 and p8 appear to be unique to HTLV-1, and p11 is peculiar to HTLV-2.

p21Rex and tRex are truncated forms of Rex-1 and Rex-2, respectively, which lack the *N*-terminal arginine-rich NLS and are therefore incapable of binding the RXRE. HTLV-2 tRex is detected as four main isoforms of 22, 20, 18, and 17 kDa which differ in the initiation codon usage and phosphorylation status (Ciminale et al., 1997). The tRex proteins were shown to inhibit Rex-2 function (Ciminale et al., 1997), an activity that might favor latent infection. Although one study indicated that p21rex acts as a repressor of full-length Rex (Heger et al., 1999), this finding was not supported by other studies (Ciminale et al., 1997; Bai et al., 2012).

HTLV-1 p30Tof and HTLV-2 p28 are important for viral propagation in animal models (Silverman et al., 2004; Yamamoto et al., 2008; Valeri et al., 2010). Both proteins sequester the tax/rex mRNA in the nucleus, an effect that may result in reduced viral expression and latency (Nicot et al., 2004; Younis et al., 2004). p30Tof was also shown to interact with the RNA-binding domain of Rex and thereby interfere with its binding to the RXRE (Sinha-Datta et al., 2007; Bai et al., 2010) and to inhibit the expression of Toll-like receptor 4 (Datta et al., 2006), suggesting a role in the innate immune response. These two properties have not been reported for p28. By interacting with the co-activator CBP/p300 (Zhang et al., 2000, 2001), p30Tof also affects Tax-mediated viral expression and transcription of cellular genes involved in T-cell activation and apoptosis (Michael et al., 2004; Taylor et al., 2009). Interestingly, p28 does not appear to affect CBP/p300-mediated transcription. Both p30Tof and p28 are targeted to the nucleus, but only p30Tof shows evident accumulation in the nucleoli (Ciminale et al., 1995; D’Agostino et al., 1997). Interestingly, HTLV-2 p28 and tRex are expressed at higher levels compared to their HTLV-1 counterparts p30/Tof and p21Rex, suggesting a higher propensity of HTLV-2 for latency compared to HTLV-1 (Bender et al., 2012).

p13 corresponds to the C-terminal 87 amino acids of p30Tof (Koralnik et al., 1992), and it is localized mainly in the mitochondrial inner membrane (Ciminale et al., 1999; D’Agostino et al., 2002) and in part to the nucleus (Silic-Benussi et al., 2010b; Andresen et al., 2011). Using a rabbit animal model, Hiraragi et al. (2006) showed that p13 is required for viral infectivity *in vivo*. p13 increases mitochondrial permeability to K^+^ and activates the electron transport chain, resulting in increased mitochondrial reactive oxygen species (ROS) production (Silic-Benussi et al., 2009). While p13 has a mitogenic effect in normal resting T cells, which have low ROS levels, the protein induces death of transformed T-cells, which are characterized by a high ROS setpoint (Ciminale et al., 1999; D’Agostino et al., 2005; Hiraragi et al., 2005; Silic-Benussi et al., 2004, 2010a,b). So far no HTLV-2 homologue of HTLV-1 p13 has been identified.

HTLV-1 p12 and its HTLV-2 homologue p10 are coded by the x-I ORF. p12 localizes in the endoplasmic reticulum (ER) and in the Golgi apparatus, where it reduces the expression of the β and γ_c_ chains of the interleukin-2 receptor (IL-2R, Mulloy et al., 1996) and of MHC-I, thus hindering lysis of HTLV-1-infected cells by CTL (Johnson et al., 2001). p12 also activates STAT-5, which provides a mitogenic signal to T cells (Nicot et al., 2001) and interacts with calreticulin and calnexin (Ding et al., 2001), resulting in incresased Ca^2+^ release from the ER (Ding et al., 2002) and activation of NF-AT, a mitogenic pathway in T-cells (Albrecht et al., 2002; Kim et al., 2003). Within the ER, p12 is cleaved into an 8-kDa protein (p8) which trafficks to the immunological synapse and favours T-cell anergy. p8 also increases cell-to-cell viral transmission through the formation of intercellular conduits (Van Prooyen et al., 2010a,b). In analogy to p12, HTLV-2 p10 was shown to bind the MHC heavy chain; however, p10 does not bind the IL2R β chain or the 16-kDa subunit of the vacuolar H+ ATPase (Johnson et al., 2000). No homologue of p8 has been described in HTLV-2.

A recent study from Valeri et al. (2010), showed that p12 is required for *in vivo* propagation in macaques but not in rabbits.

The x-V ORF of HTLV-2 codes for p11 from a doubly spliced transcript that also codes for p10 (Ciminale et al., 1995). Aside from its ability to bind to MHC heavy chain (Johnson et al., 2000), nothing is known about the function of this protein. HTLV-1 also possesses an x-V ORF, but it does not appear to produce a p11 homologue. The function of p11 is still unclear.

## HBZ AND APH-2

The structure and function of HBZ and APH-2 were recently reviewed (Barbeau et al., 2013). HBZ is a nuclear transcriptional factor (Gaudray et al., 2002), able to interact with ATF/CREB proteins through its basic zipper (bZIP) domain causing the inhibition of its DNA-binding activity (Hivin et al., 2006); this effect also influences the ability of Tax-1 to activate HTLV-1 transcription (Halin et al., 2009). APH-2 is likewise able to inhibit Tax-2-mediated transcription but its repressive activity is weaker than that of HBZ (Halin et al., 2009). This difference may be ascribed to the presence in HBZ but not in APH-2, of a transcriptional activation domain within its *N*-terminal region that mediates its interaction with the KIX domain of p300/CBP, thus competing for its binding to Tax-1 (Clerc et al., 2008).

HBZ also deregulates several cellular pathways including c-Jun and JunD, FoxP3, NF-κB, TGF-β and Wnt (Barbeau et al., 2013). In addition, HBZ, may enhance its own expression by controlling the transcriptional activity of JunD (Gazon et al., 2012). Very little is known about the regulation of APH-2 expression in infected cells. However, unlike HBZ, APH-2 enhances the transcriptional activity of c-Jun family proteins (Marban et al., 2012).

The role of HBZ in T-cell transformation is supported by the finding that while Tax-1 expression is often repressed in ATL cells and appears to be dispensable for the late stages of leukemogenesis, HBZ is constitutively expressed in most ATL cases (Satou et al., 2006). Transgenic mice expressing HBZ in CD4+ T cells develop T-cell lymphomas and systemic inflammation that are reminiscent of ATL and HAM/TSP (Satou et al., 2011).

Although HTLV-2 is not causally linked to leukemia or lymphoma, it has been associated with lymphocytosis in infected patients (Bartman et al., 2008). This is consistent with the observation that APH-2 is detected in most PBMCs of HTLV-2-infected patients (Halin et al., 2009) and that its expression is well correlated to proviral DNA load (Douceron et al., 2012). Interestingly, it was observed that the APH-2 mRNA, similarly to HBZ, accumulates in the nucleus (Bender et al., 2012). Barbeau et al. (2013) have recently proposed a model to explain the different effects of HBZ and APH-2 on T-cell proliferation. Both HBZ and of APH-2 can suppress Tax expression, thus favoring evasion of the immune response. In addition, HBZ can stimulate its own expression, inhibit Tax-1-dependent viral expression and induce T-cell proliferation, which can lead to ATL. APH-2 is unable to induce cell proliferation and only partially down-regulates Tax-2 expression.

Although dispensable for the HTLV-1 infection and immortalization of T lymphocytes *in vitro*, studies conducted on a rabbit animal model suggest that HBZ enhances HTLV-1infectivity and persistence *in vivo* (Arnold et al., 2006). Furthermore, HBZ transgenic mice develop systemic inflammation and a CD4+ T-cell neoplasm that is reminiscent of ATL (Satou et al., 2011). Interestingly, in contrast to HBZ, HTLV-2 APH-2 appears to be dispensable for viral infection and persistence in a rabbit animal model (Yin et al., 2012), suggesting a functional divergence of the *in vivo* function of the HTLV-1 and HTLV-2 antisense proteins.

## TROPISM AND CLONALITY

The preferential cellular tropism of HTLV-1 for CD4+ T cells and of HTLV-2 for CD8+ T cells is still not clearly understood. It appears that the two viruses make different use of the heparan sulfate proteoglycans to enter T cells (Jones et al., 2006), although recent evidence obtained *in vivo* rabbit model indicates that the apparent tropism for CD4+ or CD8+ cells mainly reflects preferential clonal expansion of infected cells (Kannian et al., 2012). Both HTLV-1 and HTLV-2 are capable of inducing clonal expansion of infected cells *in vivo* (Wattel et al., 1995; Cimarelli et al., 1996). A detailed analysis in asymptomatic carriers, TSP/HAM and ATLL patients revealed that the genomic integration site and transcriptional orientation of the provirus are important factors for determining clonal abundance *in vivo* (Gillet et al., 2011).

A very recent study demonstrated that, contrary to previous hypotheses, HTLV-2-infected individuals have a small number of highly expanded CD8+ T-cell clones, suggesting that HTLV-2 may be subjected to a more strict clonal selection than HTLV-1 in healthy carriers. These data suggest that selective clonal proliferation is more directly responsible for determining the viral burden of HTLV-2 than it does for HTLV-1 (Melamed et al., 2014). In contrast to observations made for HTLV-1, the environment surrounding the integration sites does not seem to have a substantial impact on the expansion of HTLV-2-infected clones (Melamed et al., 2014).

The presence of few very abundant HTLV-2-infected clones is apparently reminiscent of the profile found in ATLL patients. However, the fact that HTLV-2 is not causally linked to a T-cell malignancy suggests that clonal abundance and heterogeneity may not *per se* constitute a determinant of malignant transformation and clinical outcome of HTLV infection (Melamed et al., 2014).

## CONCLUSIONS

From the comparative analysis on the functional properties of HTLV-1 and HTLV-2 reported above, the following major differences can be outlined: (i) HTLV-2 is characterized by a more abundant expression of gene products that may favor viral latency (i.e., p28 and tRex); (ii) Tax-1 presents a PDZ binding motif, which is absent from Tax-2 and allows interaction with host factors regulating the cell cycle and proliferation; in addition, Tax-2 is unable to activate the non-canonical NF-κB pathway; (iii) *Cis*-acting inhibitory elements acting at the level of RNA stability are present within HTLV-1 whereas they have not been described in HTLV-2; (iv) while the truncated forms of Rex-2 were shown to inhibit Rex function, the HTLV-1 homologue p21Rex might lack this activity; (v) HTLV-1 expresses p13 and p8, which have not been described in HTLV-2; (vi) HTLV-2 expresses p11, which does not seem to have a homologue in HTLV-1; (vii) unlike APH-2, HBZ presents a basic zipper domain, as well a transcriptional activation domain, which mediate its capacity to enhance its own expression and deregulate several cellular pathways; (viii) HTLV-2 shows *in vivo* tropism for CD8+ T cells and induces expansion of a relatively small number of highly abundant clones.

In spite of the evidence accumulated so far on the similarities and differences between HTLV-1 and HTLV-2, it is not yet clear why only HTLV-1 causes a T-cell malignancy. While Tax-1 and HBZ induce T-cell lymphomas when expressed as transgenes in animal models, the *in vivo* transforming activities of Tax-2 and APH-2 have not been investigated. Aside from the NF-κB pathway, not much information is available regarding the interactions of Tax-2 with cellular pathways known to be engaged by Tax-1. The fact that HTLV-2, contrary to HTLV-1, is characterized by an oligoclonal proliferative distribution in asymptomatic hosts clearly indicates that the etiology of malignant transformation by HTLV-1 cannot be uniquely attributed to this phenomenon.

## Conflict of Interest Statement

The authors declare that the research was conducted in the absence of any commercial or financial relationships that could be construed as a potential conflict of interest.
